# Heparin-Binding Protein Stratifies Mortality Risk Among Ugandan Children Hospitalized With Respiratory Distress

**DOI:** 10.1093/ofid/ofae386

**Published:** 2024-07-08

**Authors:** Hridesh Mishra, Núria Balanza, Caroline Francis, Kathleen Zhong, Julie Wright, Andrea L Conroy, Robert O Opoka, Quique Bassat, Sophie Namasopo, Kevin C Kain, Michael T Hawkes

**Affiliations:** Sandra A. Rotman Laboratories, Sandra Rotman Centre for Global Health, University Health Network–Toronto General Hospital, Toronto, Ontario, Canada; ISGlobal, Hospital Clínic–Universitat de Barcelona, Barcelona, Spain; Sandra A. Rotman Laboratories, Sandra Rotman Centre for Global Health, University Health Network–Toronto General Hospital, Toronto, Ontario, Canada; Sandra A. Rotman Laboratories, Sandra Rotman Centre for Global Health, University Health Network–Toronto General Hospital, Toronto, Ontario, Canada; Sandra A. Rotman Laboratories, Sandra Rotman Centre for Global Health, University Health Network–Toronto General Hospital, Toronto, Ontario, Canada; Tropical Disease Unit, Division of Infectious Diseases, Department of Medicine, University of Toronto, Toronto, Ontario, Canada; Department of Pediatrics, Indiana University School of Medicine, Indianapolis, Indiana, USA; Medical College, East Africa, Aga Khan University, Nairobi, Kenya; Department of Pedatrics, Global Health Uganda, Kampala, Uganda; ISGlobal, Hospital Clínic–Universitat de Barcelona, Barcelona, Spain; Department of Pediatrics, Centro de Investigação em Saúde de Manhiça, Maputo, Mozambique; Department of Pediatrics, ICREA, Barcelona, Spain; Pediatrics Department, Hospital Sant Joan de Déu, Universitat de Barcelona, Barcelona, Spain; CIBER de Epidemiología y Salud Pública, Instituto de Salud Carlos III, Madrid, Spain; Department of Paediatrics, Kabale Regional Referral Hospital, Kabale, Uganda; Sandra A. Rotman Laboratories, Sandra Rotman Centre for Global Health, University Health Network–Toronto General Hospital, Toronto, Ontario, Canada; Tropical Disease Unit, Division of Infectious Diseases, Department of Medicine, University of Toronto, Toronto, Ontario, Canada; Department of Experimental Therapeutics, University Health Network–Toronto General Hospital, Toronto, Ontario, Canada; Faculty of Medicine, University of Toronto, Toronto, Ontario, Canada; Department of Pediatrics, BC Children's Hospital, University of British Columbia, Vancouver, British Columbia, Canada

**Keywords:** heparin-binding protein, mortality, pneumonia, prognostic marker, risk stratification

## Abstract

**Background:**

Current prognostic tools do not reliably and objectively identify children with pneumonia at risk of a severe or life-threatening episode. Heparin-binding protein (HBP) is a host immune protein that is released in response to infection. We hypothesized that measuring HBP concentrations at hospital admission could help risk-stratify children with pneumonia and identify those at higher risk of an adverse prognosis.

**Methods:**

We evaluated the prognostic accuracy of HBP for predicting in-hospital mortality among children with respiratory distress, and whether HBP could improve the accuracy of validated composite clinical severity scores.

**Results:**

Of 778 Ugandan children under 5 years of age and presenting with clinically defined pneumonia, 60 (7.7%) died during hospital admission. HBP concentrations at presentation were significantly higher in children with fatal outcomes (median, 76 ng/mL [interquartile range {IQR}, 41–150]) compared to children who survived (median, 31 ng/mL [IQR, 18–57]) (*P* < .001). Children with HBP >41 ng/mL on admission had an elevated risk of death (hazard ratio, 5.3 [95% confidence interval {CI}, 2.9–9.5]; *P* < .0001). In receiver operating characteristic (ROC) curve analysis, HBP concentrations distinguished between fatal and nonfatal outcomes (area under the ROC curve, 0.75 [95% CI, .66–.84]) and significantly improved the prediction provided by the Respiratory Index of Severity in Children, a composite clinical severity score (*P* = .0026).

**Conclusions:**

Measuring HBP at presentation could help identify children at risk of severe and fatal pneumonia. Adding HBP to clinical scores could improve the recognition and triage of children with pneumonia at risk of death.

Pneumonia is a leading cause of childhood mortality resulting in >700 000 annual deaths globally [1]. Mortality remains disproportionately high in resource-constrained settings where risk factors including malnourishment, coexisting infections such as human immunodeficiency virus (HIV), nonexclusive breastfeeding, crowding, lack of immunization, and indoor air pollution contribute to a higher burden of severe and fatal pneumonia [2–5].

Early triage with accurate tools could enhance early intervention and thus reduce pneumonia-associated deaths; however, there are few tools currently capable of reliably identifying children at risk of severe and fatal pneumonia [6]. Available prognostic tools include composite clinical severity scores (eg, the Respiratory Index of Severity in Children [RISC], Signs of Inflammation in Children That Kill [SICK], and the Lambaréné Organ Dysfunction Score [LODS]), pulse oximetry [7], or laboratory markers (eg, lactate, C-reactive protein [CRP], and procalcitonin [PCT]) [8–10]. Clinical scores are subjective, complicated to compute at the bedside, and are often not applied in clinical practice [11, 12]. Laboratory markers CRP and PCT are more commonly used in high-income countries and may not be available for use in clinical practice in low- and middle-income countries. CRP and PCT have been extensively used in clinical practice for evaluating inflammatory responses [13–15], and these markers' usefulness have been reported in children with complicated pneumonia. However, their predictive accuracy for fatal outcomes is poor. For example, in a previous study, the area under the receiver operating characteristic curve (AUROC) for CRP and PCT was 0.56 and 0.65, respectively [9].

The World Health Organization (WHO) provides several clinical algorithms for triage and classification of respiratory disease in low-resource environments. These include the Integrated Management of Childhood Illness (IMCI) algorithm [16], Emergency Triage Assessment and Treatment Plus [17], and the Pocket Book of Hospital Care for Children [18]. IMCI, though intended for community health workers and outpatient care, provides a convenient scheme. Cases of cough or difficulty breathing are classified as cough or cold; pneumonia; or severe pneumonia/very severe disease. The classification depends upon the presence of tachypnea, chest indrawing, and “danger signs” (not eating or drinking, vomiting everything, altered consciousness, convulsions, stridor at rest, or severe malnutrition). Since this clinical algorithm depends on nonspecific signs, it has limited ability to distinguish between the etiologies of respiratory illness [19]. For example, in addition to bacterial and viral lower respiratory tract infection (LRTI), the IMCI criteria may misclassify children with malaria with respiratory distress (MRD), associated with pulmonary edema or hyperlactatemia, as “pneumonia” or “severe pneumonia.” Likewise, patients with sepsis may present with respiratory distress and fall under the heterogeneous category “severe pneumonia/very severe disease.” In the present report, we refer to the constellation of IMCI criteria (cough or difficulty breathing, tachypnea, or chest indrawing, with or without danger signs) as “respiratory distress” to avoid ambiguity.

Heparin-binding protein (HBP) belongs to the family of serine proteases. It is released from azurophilic granules of neutrophils upon stimulation during inflammatory responses [20]. HBP is a potent inducer of vascular leakage, binding to glycosaminoglycans on the endothelial cell surface and triggering endothelial cell contraction and tight junction redistribution [21]. Thus, HBP may participate in the pathologic processes leading to alveolar consolidation in pneumonia as well as pulmonary edema in malaria. HBP has been reported to be a potential marker of severe pneumonia and progression to sepsis, but data regarding its prognostic performance remain limited [21–23]. For the pediatric population, previous publications investigated the utility of HBP to discriminate between pneumonia and complicated pneumonia in critically ill children [21, 22]. However, no previous studies have evaluated the prognostic accuracy of HBP for in-hospital mortality in children presenting with pneumonia.

Here we test the hypothesis that HBP measured at hospital admission will identify Ugandan children at risk of fatal outcome and will improve the predictive accuracy of clinical scoring systems designed to triage febrile children. Because of the heterogeneity of clinically defined pneumonia and respiratory distress, we also examined subgroups including LRTI, MRD, and sepsis.

## MATERIALS AND METHODS

### Study Design

This was a secondary analysis of a prospective cohort study that recruited children aged 2–60 months with febrile syndrome hospitalized between February 2012 and August 2013 at Jinja Regional Referral Hospital, Uganda [9].

### Study Cohort

**Figure 1.**
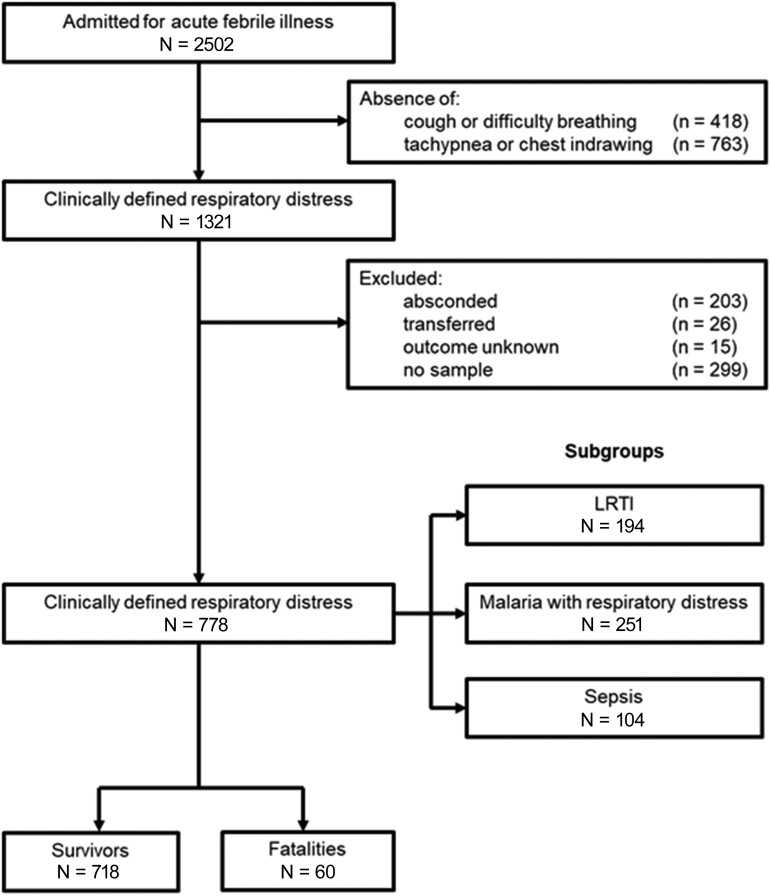
Flowchart of study participants. Clinically defined respiratory distress followed the World Health Organization Integrated Management of Childhood Illness algorithm (cough or difficulty breathing, with tachypnea or chest indrawing). Abbreviation: LRTI, lower respiratory tract infection.

Inclusion criteria for the parent study were (1) age 2–60 months; (2) parental report of fever within <48 hours or axillary temperature at presentation >37.5°C; and (3) hospitalization warranted according to the admitting physician's judgment. Additional inclusion criteria for the present analysis were (4) cough or difficulty breathing; (5) tachypnea or chest indrawing; (6) known outcome (discharge or death); and (7) plasma sample available for HBP assay. A trial flow diagram is shown in Figure 1.

### Study Procedures

Clinical investigations at the time of hospitalization included malaria diagnostics, peripheral oxygen saturation, lactate (Lactate Scout analyzer, Sports Resource Group, Minneapolis, Minnesota), and a rapid HIV test (Alere Determine, Orgenics, Yavne, Israel). With respect to malaria diagnosis, we used a 3-band rapid diagnostic malaria test (mRDT) capable of detecting histidine-rich protein 2 (HRP2) and parasite lactate dehydrogenase (pLDH) (First Response Malaria Ag HRP2/pLDH Combo Rapid Diagnostic Test, Premier Medical Corporation, India). In addition, Field-stained peripheral blood thick smears were examined by light microscopy. Parasitemia was graded using the “plus” scale: + (1 to 9 trophozoites in 100 fields); ++ (1 to 10 trophozoites in 10 fields); +++ (1 to 10 trophozoites per field); ++++ (>10 trophozoites per field). The semi-quantitative grade was used to estimate the parasite density since “+++” or “++++” correspond to a parasite density of approximately 5000 and 50 000 parasites/µL, respectively [24]. Of note, among children with respiratory distress with a circulating parasite density >2500 parasite/µL, the malaria attributable fraction is >90% [25].

### Quantification of Markers of Immune Response

Ethylenediaminetetraacetic acid–anticoagulated plasma was collected at hospital presentation and stored at −80°C until tested. HBP was quantified by enzyme-linked immunosorbent assay (ELISA) (Invitrogen Canada, Burlington, Ontario). To define “elevated” HBP, we used receiver operating characteristic (ROC) curve analysis (described below) to compute the Youden index, a cutoff associated with optimal sensitivity and specificity for prediction of mortality. CRP was quantified by ELISA and PCT was quantified using a Luminex multiplex platform with reagents from R&D Systems (data published previously [9]). Protein concentrations below the limit of detection were assigned a value of one-third of the lowest point on the standard curve. Assays were performed blinded to the study endpoint.

### Clinical Definitions and Composite Severity Scores

Respiratory distress was defined as (1) cough or difficulty breathing; and (2) age-specific tachypnea or chest indrawing [16]. Tachypnea was defined as respiratory rate ≥50 breaths/minute if <12 months old or ≥40 breaths/minute if ≥12 months old [16]. General danger signs were defined as convulsions; altered consciousness (“alert, voice, pain, unresponsive” scale less than “alert”); inability to drink or feed; vomiting everything; severe malnutrition (weight-for-age z-score < −3 standard deviations below the mean); or stridor at rest. LRTI was defined as respiratory distress without evidence of malaria (negative mRDT for both HRP2 and pLDH, and negative microscopy). MRD was defined as respiratory distress together with a semi-quantitative parasitemia grade of “+++” or “++++” on microscopy, which represents a parasite density >2500 parasites/µL and a malaria attributable fraction >90%. Sepsis was diagnosed by the admitting clinician. With respect to composite clinical severity scores, RISC was calculated as previously described for the overall cohort and the subgroup of patients with LRTI [26]. LODS, which is based on the presence of prostration, coma, and deep breathing, was computed for patients with MRD [11, 27, 28]. SICK was computed as previously described for patients with sepsis, based on the following clinical variables: age, temperature, heart rate, respiratory rate, systolic blood pressure, oxygen saturation, capillary refill time, and level of consciousness [29].

### Sample Size Calculation

A standard sample size calculation showed that we would need 568 patients to detect a difference in mean HBP concentration in fatal compared to nonfatal cases, with 80% power at the α = .05 level of significance. This calculation assumed mortality of 3.8% in the cohort [9], a mean HBP level of 40 ng/mL in survivors (standard deviation, 65 ng/mL), and a mean HBP level in fatal cases of 80 ng/mL [30].

### Statistical Analysis

Statistical analysis was performed using Stata version 15 (StataCorp, College Station, Texas). The primary outcome was in-hospital mortality. Descriptive data were summarized as number (%) and median (interquartile range [IQR]) and compared using the χ^2^ or Wilcoxon rank-sum test, as appropriate. Correlations between continuous variables were analyzed using Kendall rank correlation coefficient (tau-b, τ). ROC curve analysis was used to assess the ability of HBP to discriminate between fatal and nonfatal cases. The method described by DeLong and colleagues was used to compare the AUROC [31]. The discriminatory power of the combination of HBP and clinical scores was determined using a multivariable logistic regression model with mortality as the binary dependent variable, together with HBP and the clinical score as independent variables. Model predictions were then used to create the ROC curve. To determine the statistically optimum cutoff plasma concentration of HBP, we used the Youden index. Kaplan-Meier survival curves were constructed for patients with HBP levels above and below the cutoff, and the time to death was compared using the log-rank test. The hazard ratio (HR) with 95% confidence interval (CI) was determined using a Cox proportional hazard model.

### Ethical Considerations

The accompanying caregiver provided informed written consent. Ethical approval was obtained from the School of Biomedical Sciences Research Ethics Committee (Makerere University, Kampala, Uganda, REC Protocol # REF 2011–255), the Ugandan National Council for Science and Technology, and the University Health Network Research Ethics Board (Toronto, Canada, REB12-0039-AE). The trial was registered at ClinicalTrials.gov (NCT04726826).

## RESULTS

### Description of Cohort

The parent study enrolled 2502 children. Of these, 1321 (53%) had respiratory distress, and 778 (58%) had known outcome and plasma specimens available for analysis (survivors = 718 and nonsurvivors = 60) (Figure 1). A comparison of characteristics of patients included in the analysis and those with a missing sample is shown in Supplementary Table 1. Characteristics of the included patients are shown in Table 1, disaggregated by outcome. Completeness of data is assessed in Supplementary Table 2. The overall median HBP plasma concentration in the cohort was 32 ng/mL (IQR, 18–61).

**Table 1.** Comparison of Demographics and Clinical Characteristics Between Survivors and Fatalities

Characteristic	Overall (n = 778)	Survivors (n = 718)	Fatalities (n = 60)	*P* Value
Demographic characteristics				
Age, mo	16 (9–24)	16 (9–24)	15.5 (8.5–24)	.59
Female sex	344 (44)	318 (44)	26 (43)	.71
Clinical characteristics				
Weight, kg	9.0 (7.5–10)	9.0 (7.7–10)	8.0 (6.0–10)	.020
Height, cm	72 (65–80)	72 (65–80)	71 (60–79)	.081
Severely underweight^a^	88 (11)	75 (10)	13 (22)	.**016**
Severe wasting^b^	35 (4.5)	31 (4.3)	4 (6.7)	.218
Severe stunting^c^	294 (38)	264 (37)	30 (50)	.**035**
MUAC <11.5 cm	35 (5.0)	25 (3.9)	10 (21)	**<**.**001**
Tachycardia	575 (74)	536 (75)	39 (65)	.**004**
Tachypnea	778 (100)	718 (100)	60 (100)	>.99
Hypoxemia (SpO_2_ <92%)	78 (10)	56 (7.9)	22 (39)	**<**.**001**
Level of consciousness				**<**.**001**
Alert	587 (77)	573 (81)	14 (24)	
Voice	35 (5)	33 (5)	2 (3)	
Pain	106 (14)	80 (11)	26 (44)	
Unresponsive	36 (5)	19 (3)	17 (29)	
Danger signs				
Unable to eat/drink	227 (29)	177 (25)	50 (83)	**<**.**001**
Vomiting everything	257 (33)	232 (32)	26 (44)	.064
Altered consciousness	165 (21)	125 (17)	40 (67)	**<**.**001**
Convulsions	139 (18)	121 (17)	18 (30)	**<**.**001**
RISC	2 (0–3)	1 (0–2)	3 (3–4)	**<**.**001**
LODS	1 (0–2)	1 (0–2)	3 (2–3)	**<**.**001**
SICK	2 (1–3)	2 (1–3)	4 (3–5)	**<**.**001**
Diagnoses				
LRTI	194 (25)	172 (24)	22 (37)	.**026**
*Plasmodium falciparum* detected^d^	441 (58)	416 (59)	25 (43)	.**017**
MRD^e^	251 (32)	243 (34)	8 (13)	.**001**
Sepsis	104 (13)	80 (11)	24 (40)	**<**.**001**
HIV	18 (2)	13 (12)	5 (8)	.**001**
Biomarkers				
Heparin-binding protein, ng/mL	32 (18–61)	31 (18–57)	76 (41–150)	**<**.**001**
Lactate, mmol/L	2.9 (2.0–7.2)	2.9 (2.0–6.4)	9.8 (2.9–17.3)	**<**.**001**
Procalcitonin, ng/mL	4.4 (1.0–14)	2.7 (0.77–9.4)	8.0 (2.2–26)	**<**.**001**
C-reactive protein, µg/mL	120 (47–210)	110 (41–190)	170 (58–290)	.**038**

Data are presented as median (interquartile range) or No. (%). Values in bold represent statistically significant differences.

Abbreviations: HIV, human immunodeficiency virus; LODS, Lambaréné Organ Dysfunction Score; LRTI, lower respiratory tract infection; MRD, malaria with respiratory distress; MUAC, mid-upper arm circumference; RISC, Respiratory Index of Severity in Children; SICK, Signs of Inflammation in Children That Kill; SpO_2_, oxygen saturation.

^a^More than 3 standard deviations below the mean weight-for-age, based on World Health Organization (WHO) growth charts.

^b^More than 3 standard deviations below the mean weight-for-length/height, based on WHO growth charts.

^c^More than 3 standard deviations below the mean length/height-for-age, based on WHO growth charts.

^d^Positive malaria rapid diagnostic test or microscopy at any parasite density.

^e^High parasite density was used to select a subgroup with high malaria attributable fraction.

One hundred ninety-four patients tested negative for malaria (25%) and likely had bacterial or viral LRTI as the cause of their fever and respiratory distress (LRTI subgroup). Five hundred seventy-four patients tested positive for malaria (74%) and 251 (32%) had a semi-quantitative parasite density of “+++” or “++++” on microscopy, corresponding to a group with high malaria attributable fraction (MRD subgroup). One hundred four patients (13%) had a clinical diagnosis of sepsis (sepsis subgroup).

### HBP Is Associated With Hypoxemia and Disease Severity

**Figure 2.**
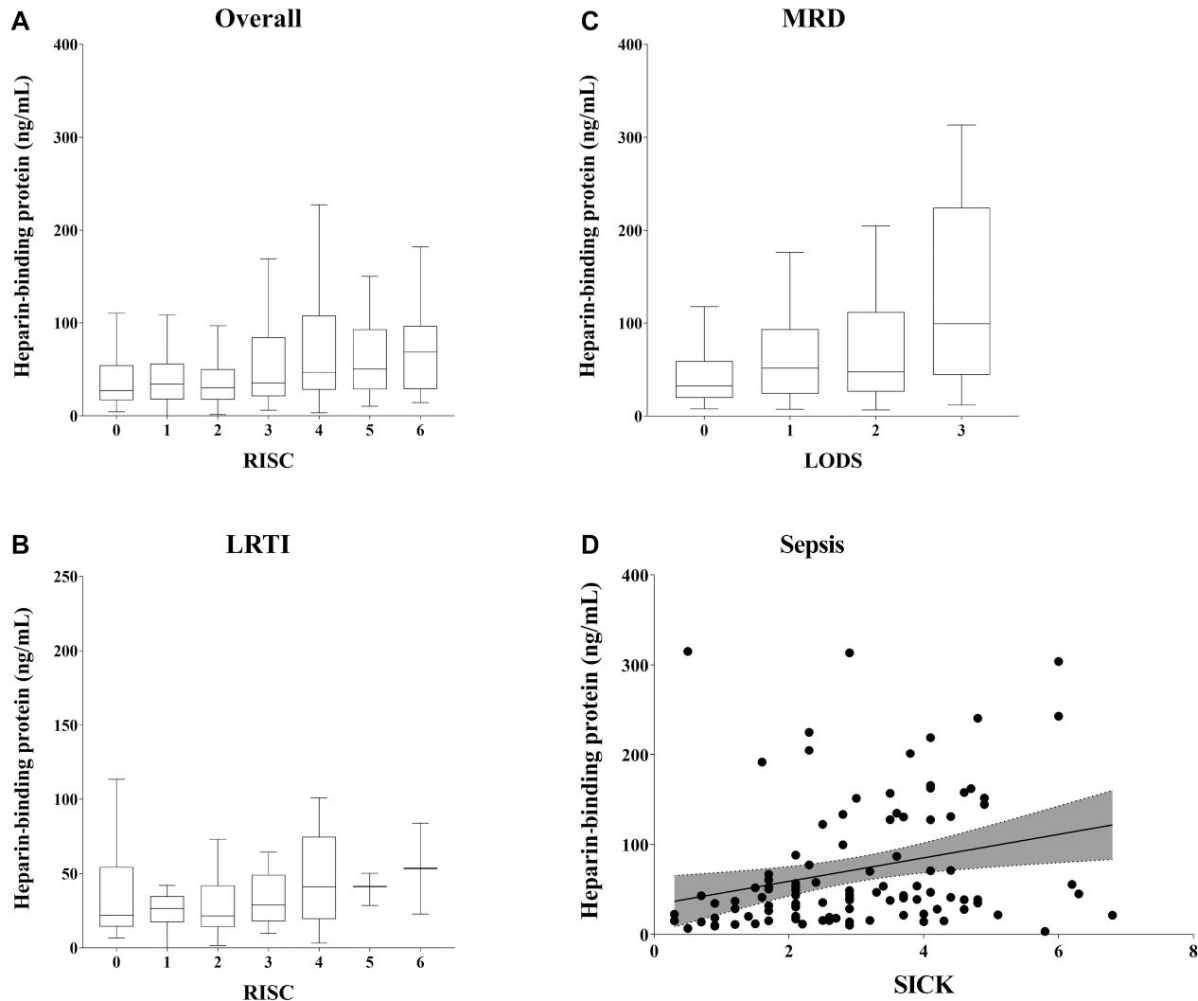
Heparin-binding protein (HBP) was associated with disease severity. *A*, In the overall cohort of children with respiratory distress, levels of HBP increased with increasing Respiratory Index of Severity in Children (RISC) (Kendall rank correlation coefficient τ = 0.11, *P* < .0001). *B*, In the subgroup of children with lower respiratory tract infection (LRTI), levels of HBP increased with increasing RISC (τ = 0.12, *P* = .032). *C*, In the subgroup of children with malaria with respiratory distress (MRD), levels of HBP increased with increasing Lambaréné Organ Dysfunction Score (LODS, τ = 0.14, *P* = .00034). *D*, Levels of HBP increased with increasing Signs of Inflammation in Children That Kill (SICK) score (τ = 0.24, *P* = .00039).

The median HBP level in hypoxemic patients (SpO_2_ <92%) was 45 ng/mL (IQR, 21–120) compared to 31 ng/mL (IQR, 18–57) in patients with SpO_2_ ≥92% (*P* = .0024). In the overall cohort of patients with respiratory distress, the HBP concentration increased with increasing disease severity, as measured by the RISC (τ = 0.11, *P* < .0001; Figure 2*A*). In the LRTI subgroup, the correlation between HBP and RISC was similar (τ = 0.12, *P* = .032; Figure 2*B*). In the MRD subgroup, the correlation between HBP and LODS was τ = 0.14 (*P* = .0034; Figure 2*C*). In the sepsis subgroup, the correlation between HBP and SICK was τ = 0.24 (*P* = .00039; Figure 2*D*). Of note, in a sensitivity analysis, exclusion of children living with HIV did not affect the correlation between RISC and HBP (Supplementary Table 3).

### HBP Is Correlated With Other Markers of Systemic Inflammation Used in Clinical Practice

Lactate has prognostic significance in children with pneumonia and malaria with respiratory distress [32, 33]. Likewise, markers of inflammation used in clinical practice (CRP and PCT) may predict adverse outcomes. We observed statistically significant correlations between HBP and these 3 markers (Table 2).

**Table 2.** Correlations (τ) Between Heparin-Binding Protein and Other Laboratory Markers of Inflammation Used in Clinical Practice

Laboratory Marker	Overall (n = 778)	LRTI (n = 194)	MRD (n = 251)	Sepsis (n = 102)
Lactate	0.23***	0.037	0.33***	0.16*
C-reactive protein	0.21***	0.21*	0.14	0.32**
Procalcitonin	0.23***	0.22*	0.35**	0.30*

^*^ *P* < .05; **P < .01; ***P < .001.

Abbreviations: LRTI, lower respiratory tract infection; MRD, malaria with respiratory distress.

### HBP Predicts In-Hospital Mortality

**Figure 3.**
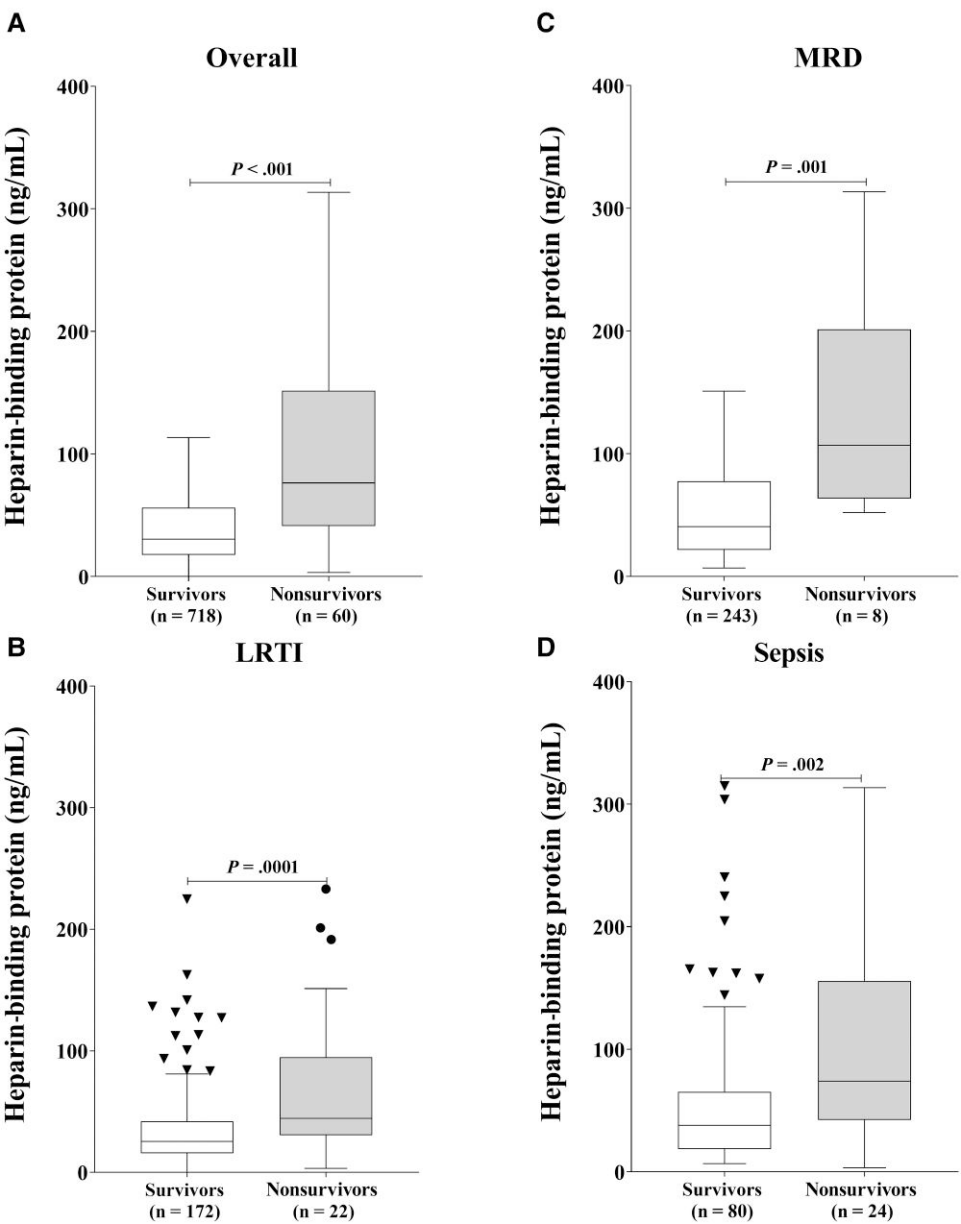
Heparin-binding protein was higher in fatal compared to nonfatal cases for overall cohort (*A*), subgroup with lower respiratory tract infection (LRTI) (*B*), subgroup with malaria with respiratory distress (MRD) (*C*), and subgroup with sepsis (*D*). ^a^Respiratory Index of Severity in Children for LRTI, Lambaréné Organ Dysfunction Score for malaria, and Signs of Inflammation in Children That Kill for sepsis. *Versus heparin-binding protein. †Versus clinical severity score.

There were 60 deaths (case fatality rate 7.7%). Fifty-six deaths (93%) occurred within 48 hours of admission. In the overall cohort, HBP concentrations were significantly higher in nonsurvivors (median, 76 ng/mL [IQR, 41–150]) than survivors (30 ng/mL [IQR, 18–56]) (*P* < .0001). Statistically significant differences in HBP between fatal and nonfatal cases were also observed in all subgroups (Figure 3).

**Figure 4.**
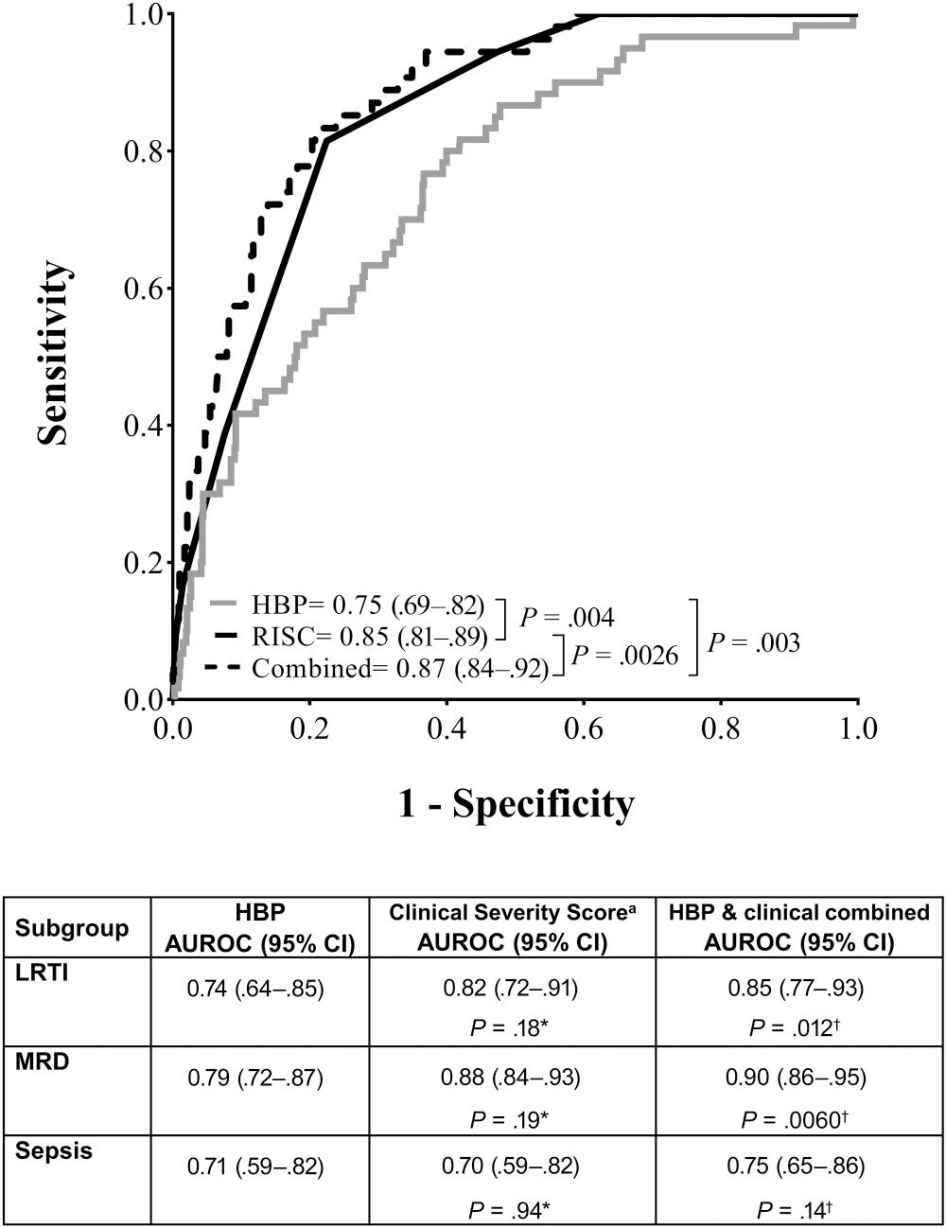
Receiver operating characteristic curve analysis for mortality prediction. The overall cohort (n = 752 after exclusion of patients with missing data for Respiratory Index of Severity in Children) is shown graphically. Subgroup analyses are shown in the table below. Abbreviations: AUROC, area under the receiver operating characteristic curve; CI, confidence interval; HBP, heparin-binding protein; LRTI, lower respiratory tract infection; MRD, malaria with respiratory distress; RISC, Respiratory Index of Severity in Children.

ROC curve analysis applied to HBP to discriminate between fatal and nonfatal outcome is shown in Figure 4. The AUROC for HBP was 0.75 (95% CI, .69–.82). The optimal cutoff (Youden index) for HBP to distinguish fatal from nonfatal outcome was 41 mg/mL. This cutoff was associated with a sensitivity of 77% (95% CI, 64%–87%) and a specificity of 63% (95% CI, 60%–67%). In comparison, the AUROC for CRP and PCT was 0.60 (95% CI, .50–.69) and 0.67 (95% CI, .58–.76), respectively. The AUROC for HBP was statistically significantly greater than for CRP (*P* = .017) but did not reach statistical significance in the comparison with PCT (*P* = .31). With respect to the composite clinical score RISC, the AUROC was 0.85 (95% CI, .81–.89). In combination with RISC, the HBP increased the AUROC to 0.87 (95% CI, .84–.92; *P* = .0026). Similarly, HBP significantly increased the AUROC when used in combination with the clinical severity score in subgroups LRTI, MRD, and sepsis (Figure 4).

**Figure 5.**
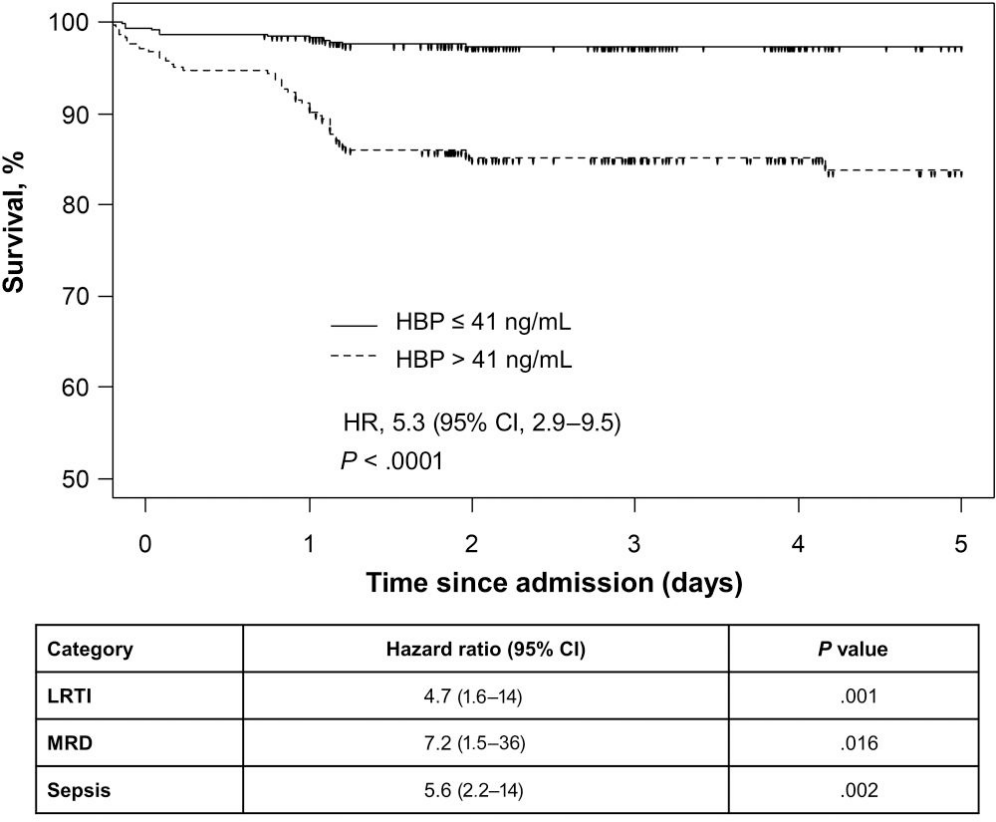
Kaplan-Meier survival curves comparing participants with admission heparin-binding protein (HBP) level above and below 41 mg/L for the overall cohort with respiratory distress (figure) and subgroups with lower respiratory tract infection (LRTI), malaria with respiratory distress (MRD), and sepsis (table). Hazard ratios (HRs) with 95% confidence interval (CI) and *P* values were calculated from Cox proportional hazard models.

Kaplan-Meier survival curves for patients with high HBP at admission (>41 ng/mL) compared to those with lower HBP (≤41 ng/mL) demonstrated a significant difference in the time to death in the overall cohort and in each subgroup (Figure 5). Elevated admission HBP increased the hazard of death by 5.3-fold (95% CI, 2.9–9.5, *P* < .0001; Figure 5). In a multivariable Cox proportional hazard model adjusting for age and sex, both RISC (adjusted HR [aHR], 2.1 [95% CI, 1.7–2.5]; *P* < .0001) and HBP (aHR, 3.3 [95% CI, 1.7–6.3]; *P* = .00026) were independent predictors of mortality. This finding remained significant after exclusion of children with HIV (Supplementary Table 3).

### Examination of Confounding Effects of Malnutrition, Sickle Cell Disease, and Treatment

We examined factors that could confound the association between HBP and mortality. Malnutrition indices were not associated with HBP level (Supplementary Table 4) but were associated with mortality (Table 1). In a multivariable logistic regression model adjusting for malnutrition indices, elevated HBP (>41 ng/mL) remained a statistically significant predictor of mortality (aOR, 6.4 [95% CI, 3.2–14], *P* < .0001; Supplementary Table 5). Sickle cell disease or hemoglobinopathy was reported in 21 patients (2.7%). After exclusion of these patients, HBP remained a statistically significant predictor of mortality (OR, 6.7 [95% CI, 3.4–14]; *P* < .0001). Antimalarial and antibiotic treatment is shown in Supplementary Table 6. Antimalarial treatment differed in fatal and nonfatal cases (Supplementary Table 6. Furthermore, admission HBP levels were different in patients treated with artesunate and cloxacillin (Supplementary Table 7). In a multivariable logistic regression model adjusting for treatment with artesunate and cloxacillin, HBP remained a statistically significant predictor of mortality (aOR, 5.4 [95% CI, 3.0–10]; *P* < .0001).

## DISCUSSION

In this study, we show that plasma concentrations of HBP at the time of presentation to healthcare facility can accurately risk-stratify children with respiratory distress and meeting criteria for IMCI pneumonia, including subgroups with LRTI, MRD, and sepsis. Previous studies have been underpowered for mortality outcomes, with few or no fatal cases [21, 22]. Our study addresses this shortcoming by investigating a large consecutive cohort of children at risk of severe and fatal outcome presenting to hospital. Another noteworthy strength of our study is the use of validated clinical scoring tools as comparators to HBP for mortality prediction [11, 27, 28].

HBP can induce vascular leakage and amplify a variety of cellular responses involved in inflammatory responses to infection. Concentrations of HBP at clinical presentation correlated with clinical severity scores, lactate, and inflammatory markers. These findings support the hypothesis that HBP is a marker and/or mediator of pathogenic processes in children with respiratory distress. Diverse etiologies of respiratory distress were likely represented in our cohort, including viral, bacterial, fungal, and *P falciparum*. Despite this diversity, HBP was consistently associated with illness severity and mortality across subgroups of children with respiratory distress. Thus, HBP appears to be intimately linked with the final common pathways of systemic inflammation and tissue hypoxia leading to death in children with pneumonia, malaria, and sepsis, irrespective of causative pathogen.

We investigated whether HBP was a clinically informative prognostic marker in children with respiratory distress. Survival analysis indicated that a high HBP at admission (>41 ng/mL) was associated with a 3.3-fold higher hazard of death in the overall cohort with respiratory distress. Likewise, high HBP was associated with a statistically significantly elevated hazard of death in subgroups with LRTI, MRD, and sepsis. Using ROC curve analysis, we examined whether HBP could add predictive value to the clinical scores RISC, LODS, and SICK for in-hospital death [11, 21–24]. In children with pneumonia, higher RISC scores indicate the need for referral and urgent care [12, 26]. In this study, the RISC score had high prognostic accuracy for death (AUROC, 0.85 [95% CI, .81–.89]) and was statistically significantly improved when used in combination with HBP (AUROC, 0.87 [95% CI, .84–.92]; *P* = .003). Furthermore, in a multivariable Cox proportional hazard model, both HBP and RISC were independent predictors of mortality, suggesting that HBP provides prognostic information beyond the clinical score alone. This suggests a possible role for HBP in clinical practice to improve triage and allocation of resources to the sickest patients. Our study adds HBP as an objective and quantitative predictor of mortality, with a proposed cutoff of 41 ng/mL in this context to achieve a balance of sensitivity and specificity.

The present study had several strengths including a large sample size with high mortality, adequately powered to assess mortality as an outcome for the entire cohort. However, there were limitations. As a secondary analysis in a subset of children included in a large prospective fever study, with archived plasma samples used for measurement of markers, the biomarker, endpoints, and methods were not specified a priori [9]. The analysis did not consider cases lost to follow-up because of abscondment or transfer to other hospitals. Radiographic imaging was not available in the parent study; thus, only using IMCI criteria may have resulted in some diagnostic misclassification. More extensive diagnostic microbiology workup would be needed to better define the etiology of LRTI and sepsis; however, this was not available in our resource-limited setting. There were some missing observations (Supplementary Table 1), though these were unlikely to affect the association between HBP and mortality. The diagnosis of sepsis, used for subgroup analysis, was based on the treating clinician's judgment and was therefore subjective. There was a prolonged interval between collection of samples and ELISA for HBP concentration in plasma; however, the sample cold chain was maintained at −80°C for this entire interval and we did not find evidence of degradation of HBP (high levels of HBP were measured and were associated with severe outcomes). There were 299 patients from the prospective cohort who were excluded from the analysis because no sample was available for quantification of HBP (Figure 1). Patients who were excluded had a lower frequency of tachypnea and tachycardia, although other characteristics were similar (Supplementary Table 1). Thus, our findings may not be representative of all patients with clinically defined pneumonia, given the overrepresentation of tachypneic and tachycardic patients in our cohort.

In conclusion, HBP, a circulating marker of host immune response to infection, was associated with increased mortality. Measuring HBP at hospital presentation may improve early triage and outcome of children with respiratory distress, including those with LRTI, MRD, and sepsis.

## Supplementary Material

ofae386_Supplementary_Data

## Supplementary Data

Supplementary materials are available at *Open Forum Infectious Diseases* online. Consisting of data provided by the authors to benefit the reader, the posted materials are not copyedited and are the sole responsibility of the authors, so questions or comments should be addressed to the corresponding author.

## Notes

***Author contributions.*** H. M. wrote the first manuscript draft, performed the laboratory assays, analyzed the data, and approved the final version. C. F. and K. Z. performed the laboratory assays and approved the final version. N. B., J. W., and Q. B. critically reviewed the manuscript and approved the final version. A. L. C. supervised the data collection, critically reviewed the manuscript, and approved the final version. R. O. O. and S. N. obtained ethical and administrative approvals, supervised the data collection, critically reviewed the manuscript, and approved the final version. K. C. K. conceived the study, obtained the funding, critically reviewed the manuscript, and approved the final version. M. T. H. designed the study, obtained ethical and administrative approvals, supervised the data collection, analyzed the data, critically reviewed the manuscript, and approved the final version.

***Financial support.*** This work was supported by the Canadian Institutes of Health Research (CIHR) (Canada Research Chair Program and CIHR Foundation Grant 148439 to K. C. K.), and by kind donations from the Tesari Foundation and Kim Kertland (to K. C. K.). ISGlobal receives support from the grant CEX2018-000806-S funded by MCIN/AEI/10.13039/501100011033, and support from the Generalitat de Catalunya through the CERCA Program. N. B. is supported by an Formació de Professorat Universitari (FPU) predoctoral fellowship from the Spanish Ministry of Universities (FPU18/04260).
