# Supplementary material for: Heparin-Binding Protein Stratifies Mortality Risk Among Ugandan Children Hospitalized With Respiratory Distress
Source: Open Forum Infect Dis. 2024 Jul 8;11(7):ofae386. doi: 10.1093/ofid/ofae386 (PMC11253034; doi:10.1093/ofid/ofae386)
Supplement: ofae386_Supplementary_Data [file ofae386_supplementary_data.docx]

**Table S1. Comparison of characteristics of patients included in the analysis with those with missing sample (no HBP measurement)**

|  | **Included in analysis**  **(n=778)** | **Missing HBP**  **(n=299)** | **P-value** |
| --- | --- | --- | --- |
| ***Demographic characteristics*** |  |  |  |
| **Age (months)** | 16 (9-24) | 15 (10.5-26.5) | 0.088 |
| **Female sex** | 344 (45) | 139 (47) | 0.52 |
| ***Clinical characteristics*** |  |  |  |
| **Weight (kg)** | 9.0 (7.5-10) | 9.1 (8.0-11) | 0.021 |
| **Height (cm)** | 72 (65-80) | 73 (67-82) | 0.0082 |
| **Severely underweight, n (%)**^a^ | 88 (12) | 24 (8.2) | 0.15 |
| **Severe wasting, n (%)**^b^ | 35 (4.7) | 12 (4.2) | 0.90 |
| **Severe stunting, n (%)**^c^ | 294 (39) | 100 (35) | 0.28 |
| **MUAC < 11.5 cm, n (%)** | 35 (5) | 9 (3.3) | 0.31 |
| **Tachycardia, n (%)** | 575 (75) | 197 (67) | 0.019 |
| **Tachypnea, n (%)** | 625 (80) | 93 (33) | <0.0001 |
| **Hypoxemia (SpO_2_ < 92%)** | 54 (7) | 18 (6.2) | 0.72 |
| **Level of consciousness, n (%)** |  |  | 0.21 |
| Alert | 587 (77) | 209 (73) |  |
| Voice | 35 (4.6) | 22 (7.7) |  |
| Pain | 106 (14) | 44 (15) |  |
| Unresponsive | 36 (4.7) | 12 (4.2) |  |
| **Danger signs** |  |  |  |
| Unable to eat/drink | 227 (29) | 80 (27) | 0.50 |
| Vomiting everything | 257 (33) | 107 (36) | 0.46 |
| Altered Consciousness | 165 (21) | 63 (21) | >0.99 |
| Convulsions | 139 (18) | 55 (18) | 0.92 |
| **RISC** | 2 (0-3) | 2 (0-3) | 0.0097 |
| **LODS** | 1 (0-2) | 1 (0-2) | 0.73 |
| **SICK** | 2.1 (1.2-2.8) | 1.7 (0.5-2.5) | <0.0001 |
| ***Diagnoses, n (%)*** |  |  |  |
| **LRTI** | 194 (25) | 81 (28) | 0.49 |
| ***P. falciparum* detected**^d^ | 574 (75) | 213 (72) | 0.49 |
| **MRD** | 251 (32) | 83 (28) | 0.17 |
| **Sepsis** | 104 (13) | 36 (12) | 0.62 |
| **HIV** | 18 (2.3) | 10 (3.4) | 0.44 |
| ***Biomarkers*** |  |  |  |
| **Lactate (mmol/L)** | 2.9 (2-7.2) | 2.9 (1.9-6.1) | 0.23 |
| **Procalcitonin (ng/ml)** | 4.4 (1.1-14) | 4.8 (0.83-15) | 0.80 |
| **C-Reactive Protein (µg/mL)** | 122 (47-214) | 145 (48.3-246) | 0.91 |
| ***Outcome*** |  |  |  |
| **Fatal outcome** | 60 (7.7) | 20 (6.8) | 0.69 |

Data presented as median (interquartile range) and n/N (%)

LODS= Lambaréné Organ Dysfunction Score, LRTI= Lower respiratory tract infection, RISC= Respiratory Index of Severity in Children, SICK= Signs of inflammation in children that kill; MRD=malaria with respiratory distress

^a^More than 3 standard deviations below the mean weight-for-age, based on WHO growth charts

^b^More than 3 standard deviations below the mean weight-for-length/height, based on WHO growth charts

^c^More than 3 standard deviations below the mean length/height-for-age, based on WHO growth charts

^d^Positive malaria rapid diagnostic test or microscopy at any parasite density.

**Table S2. Completeness of data (N=778 patients)**

|  | **Missing observations,**  **n (%)** |
| --- | --- |
| ***Demographic characteristics*** |  |
| **Age** | 0 (0) |
| **Sex** | 6 (0.77) |
| ***Clinical characteristics*** |  |
| **Weight** | 7 (0.9) |
| **Height** | 13 (1.7) |
| **MUAC** | 82 (11) |
| **Heart rate** | 8 (1) |
| **Respiratory rate** | 0 (0) |
| **Oxygen saturation** | 9 (1.2) |
| **Level of consciousness** | 14 (1.8) |
| ***Danger signs*** |  |
| **Unable to eat/drink** | 2 (0.26) |
| **Vomiting everything** | 3 (0.39) |
| **Altered Consciousness** | 11 (1.4) |
| **Convulsions** | 1 (0.13) |
| **RISC** | 26 (3.3) |
| **LODS** | 1 (0.13) |
| **SICK** | 0 (0) |
| ***Laboratory tests*** |  |
| **Malaria microscopy** | 120 (15) |
| **HIV** | 0 (0) |
| **Heparin Binding Protein** | 0 (0) |
| **Lactate** | 25 (3.2) |

**Table S3. Sensitivity analysis: effect of excluding children living with HIV (n=18)**

|  | **Including CLWH**  **(N=778)** | **Excluding CLWH**  **(N=760)** |
| --- | --- | --- |
| ***Correlation with RISC***^a^ |  |  |
| **Overall cohort** | τ=0.11  p<0.0001 | τ=0.11  p<0.0001 |
| **LRTI subgroup** | τ=0.12  p=0.032 | τ=0.11  p=0.059 |
| ***Survival analysis*** |  |  |
| **HBP*,* aHR (95%CI)** | 3.3 (1.7-6.3)  p=0.00026 | 3.9 (1.9-8.0)  p=0.00016 |
| **RISC*,* aHR (95%CI)** | 2.1 (1.7-2.5)  p<0.0001 | 2.1 (1.7-2.5)  p<0.0001 |

CLWH, children living with HIV

^a^RISC is calculated differently in children with and without HIV.

The purpose of this sensitivity analysis was to compare findings when CLWH were included or excluded from analyses that involved RISC.

*p<0.05, **p<0.01, ***p<0.0001

**Table S4. Serum HPB concentration (ng/mL) in patients according to signs of malnutrition**

|  | **Malnutrition sign present** | **Malnutrition sign absent** | **P-value** |
| --- | --- | --- | --- |
| **Severely underweight** | 29 (19-49) | 32 (18-61) | 0.21 |
| **Severe wasting** | 34 (18-52) | 31 (18-61) | 0.94 |
| **Severe stunting** | 32 (19-60) | 31 (18-61) | 0.82 |
| **MUAC < 11.5 cm** | 38 (19-58) | 32 (18-60) | 0.83 |

**Table S5. Univariable and multivariable logistic regression models using serum HPB as a predictor of mortality, with adjustment for markers of malnutrition**

|  | **Univariable**  **OR (95% CI)** | **P-value** | **Multivariable**  **aOR (95% CI)** | **P-value** |
| --- | --- | --- | --- | --- |
| **HBP>41 ng/mL** | 5.6 (3.1-11) | <0.0001 | 6.4 (3.2-14) | <0.0001 |
| **Severely underweight** | 2.4 (1.2-4.6) | 0.0084 | 1.1 (0.35-3.0) | 0.87 |
| **Severe wasting** | 1.6 (0.48-4.4) | 0.36 | 1.1 (0.25-4.1) | 0.86 |
| **Severe stunting** | 1.8 (1.1-3.2) | 0.027 | 1.3 (0.66-2.5) | 0.43 |
| **MUAC < 11.5 cm** | 6.5 (2.8-14) | <0.0001 | 5.6 (2.0-15) | 0.00090 |

**Table S6. Antimalarial and antibiotic treatment, stratified by mortality**

|  | **Entire Cohort**  **(N=778)** | **Non-fatal**  **(N=718)** | **Fatal**  **(N=60)** | **P-value** |
| --- | --- | --- | --- | --- |
| ***Antimalarials*** |  |  |  |  |
| Quinine | 507 (65) | 486 (68) | 21 (35) | <0.0001 |
| Artesunate | 65 (8.4) | 54 (7.5) | 11 (18) | 0.0077 |
| Artemether | 31 (4) | 29 (4) | 2 (3.3) | >0.99 |
| Artemether-lumefantrine (po) | 37 (4.8) | 37 (5.2) | 0 (0) | 0.11 |
| ***Antibiotics*** |  |  |  |  |
| Ceftriaxone | 479 (62) | 436 (61) | 43 (72) | 0.13 |
| Gentamicin | 227 (29) | 204 (28) | 23 (38) | 0.14 |
| Penicillin | 94 (12) | 91 (13) | 3 (5) | 0.097 |
| Ampicillin | 109 (14) | 104 (14) | 5 (8.3) | 0.26 |
| Amoxicillin | 13 (1.7) | 13 (1.8) | 0 (0) | 0.61 |
| Cloxacillin | 38 (4.9) | 33 (4.6) | 5 (8.3) | 0.33 |
| Trimethoprim-sulfamethoxazole | 5 (0.64) | 4 (0.56) | 1 (1.7) | 0.33 |
| Metronidazole | 4 (0.51) | 3 (0.42) | 1 (1.7) | 0.28 |

Data represent n (%)

**Table S7. Levels of HBP (ng/mL), according to treatment with antimalarials and antibiotics**

| **Agent** | **Treated with agent** | **Not treated with agent** | **P-value** |
| --- | --- | --- | --- |
| ***Antimalarials*** |  |  |  |
| Quinine | 32 (19-61) | 32 (18-55) | 0.78 |
| Artesunate | 45 (22-110) | 31 (18-58) | 0.0023 |
| Artemether | 43 (29-130) | 31 (18-59) | 0.02 |
| Artemether-lumefantrine (po) | 33 (17-59) | 32 (18-61) | 0.61 |
| ***Antibiotics*** |  |  |  |
| Ceftriaxone | 34 (19-63) | 30 (18-57) | 0.072 |
| Gentamicin | 29 (18-53) | 34 (19-64) | 0.051 |
| Penicillin | 30 (17-43) | 32 (19-64) | 0.14 |
| Ampicillin | 27 (17-50) | 33 (19-62) | 0.059 |
| Amoxicillin | 32 (19-60) | 32 (18-61) | 0.93 |
| Cloxacillin | 49 (26-84) | 31 (18-59) | 0.028 |
| Trimethoprim-sulfamethoxazole | 31 (9.7-39) | 32 (18-61) | 0.48 |
| Metronidazole | 37 (30-61) | 32 (18-61) | 0.66 |

Numbers represent median (IQR)
